# Youth is Prized in Medicine, Old Age is Valued in Law: Analysis of Media Narratives Over 200 Years

**DOI:** 10.2196/45855

**Published:** 2024-03-26

**Authors:** Reuben Ng, Nicole Indran

**Affiliations:** 1 Lee Kuan Yew School of Public Policy National University of Singapore Singapore Singapore; 2 Lloyd’s Register Foundation Institute for the Public Understanding of Risk National University of Singapore Singapore Singapore

**Keywords:** older professionals, ageism, media, historical analysis, reframe aging, learned professions, psychomics

## Abstract

**Background:**

This is the first study to explore how age has influenced depictions of doctors and lawyers in the media over the course of 210 years, from 1810 to 2019. The media represents a significant platform for examining age stereotypes and possesses tremendous power to shape public opinion. Insights could be used to improve depictions of older professionals in the media.

**Objective:**

This study aims to understand how age shapes the portrayals of doctors and lawyers. Specifically, it compares the difference in sentiments toward younger and older doctors as well as younger and older lawyers in the media over 210 years.

**Methods:**

Leveraging a 600-million-word corpus of American media publications spanning 210 years, we compiled top descriptors (N=478,452) of nouns related to youth × occupation (eg, younger doctor or physician) and old age × occupation (eg, older lawyer or attorney). These descriptors were selected using well-established criteria including co-occurrence frequency and context relevance, and were rated on a Likert scale from 1 (very negative) to 5 (very positive). Sentiment scores were generated for “doctor/physician,” “young(er) doctor/physician,” “old(er) doctor/physician,” “lawyer/attorney,” “young(er) lawyer/attorney,” and “old(er) lawyer/attorney.” The scores were calculated per decade for 21 decades from 1810 to 2019. Topic modeling was conducted on the descriptors of each occupation in both the 1800s and 1900s using latent Dirichlet allocation.

**Results:**

As hypothesized, the media placed a premium on youth in the medical profession, with portrayals of younger doctors becoming 10% more positive over 210 years, and those of older doctors becoming 1.4% more negative. Meanwhile, a premium was placed on old age in law. Positive portrayals of older lawyers increased by 22.6% over time, while those of younger lawyers experienced a 4.3% decrease. In the 1800s, narratives on younger doctors revolved around their participation in rural health care. In the 1900s, the focus shifted to their mastery of new medical technologies. There was no marked change in narratives surrounding older doctors from the 1800s to the 1900s, though less attention was paid to their skills in the 1900s. Narratives on younger lawyers in the 1800s referenced their limited experience. In the 1900s, there was more focus on courtroom affairs. In both the 1800s and 1900s, narratives on older lawyers emphasized their prestige, especially in the 1900s.

**Conclusions:**

Depending on the occupation, one’s age may either be seen as an asset or a liability. Efforts must be expended to ensure that older professionals are recognized for their wealth of knowledge and skills. Failing to capitalize on the merits of an older workforce could ultimately be a grave disservice not only to older adults but to society in general.

## Introduction

Due to medical advances, older adults today are much healthier than before and make up a substantial portion of the labor market. The share of individuals aged 55 years and older either working or actively seeking employment has increased significantly since the 1990s, with approximately 37 million older Americans in the workforce as of March 2021 [1]. Never in history have older people been more integral to the workforce, and projections indicate a continuation of this trend [2]. Exploring how older workers have been portrayed in the media is essential for fostering a society that values its older population. In this study, we compare the portrayals of younger and older practitioners in the legal and medical spheres over the last 210 years.

The significance of our study lies in both conceptual and practical domains. From a conceptual standpoint, this study is one of the first to examine how portrayals of older workers in the media have changed over the span of 210 years. Existing research has looked only at how older adults in general are stereotyped in the media [3]. By analyzing the portrayals of older workers over such an extensive period, this study facilitates a more comprehensive understanding of how various social, cultural, or industry-related factors may have influenced these portrayals. This will in turn allow for better contextualization of current attitudes toward older workers. From a practical standpoint, insights from this study can inform interventions aimed at combating negative age stereotypes of older workers in the media.

The theory of social constructionism maintains that reality is neither objective nor fixed, but rather is constructed through social and cultural processes [4]. As a key agent for transmitting and disseminating information, the media plays a salient role in shaping the way reality is interpreted. According to the agenda-setting theory, the media molds public opinion by determining which issues are considered important and worthy of attention [5,6]. Similarly, cultivation theory posits that repeated exposure to certain messages in the media can alter people’s attitudes and beliefs over time [7]. Given the power of the media to influence the collective conscience, it is critical to inspect the ways older workers have been depicted in the media.

Several studies have looked at how older people are portrayed in the media. Ng et al [3] discovered that age stereotypes in the United States have become more negative over the last 2 centuries. Meanwhile, some have observed that depictions of older persons in visual media have become more positive in Europe and America since the 1950s [8,9]. To date, there is a paucity of literature on how different subgroups of older people are stereotyped in the media. The existing studies have focused primarily on the portrayal of grandparents [10], with little attention dedicated to the stereotypes of older workers.

Over the decades, scholars have endeavored to dissect the nature of age stereotypes. Although there is copious evidence of a general negative bias in societal perceptions of older adults [3,11-15], age stereotypes are widely accepted as being multifaceted [16,17]. Negative age stereotypes include being frail and unfriendly, while positive ones include being warm and amiable. Psychologists have argued that the stereotypes applied to a given target tend to shift across contexts as only contextually meaningful information will be used to evaluate the individual [18,19]. This contextual malleability of stereotypes renders it a scholarly imperative to pinpoint the various circumstances in which positive and negative age stereotypes emerge.

Presently, most studies pertaining to older workers focus on age discrimination as well as stereotypes in the workplace [20,21]. Positive stereotypes of older workers class them as warm, reliable, and committed to the job [22,23], while negative ones include being less flexible and adaptable [23,24]. Meanwhile, even as younger workers are highly regarded in terms of physical ability, productivity, and creativity [23], they are often branded as being inexperienced, unreliable, and unmotivated [25].

The ways in which older workers are represented in the media can affect the public’s attitudes toward old age and in turn the health of older persons. According to stereotype embodiment theory, the assimilation of age stereotypes into one’s self-concept can affect one’s health [26]. Negative age stereotypes are linked to poorer health outcomes such as a reduced sense of self-efficacy, a higher risk of depression as well as poorer immune or cardiovascular health [26-29]. Conversely, positive age stereotypes are associated with improved functional health, well-being, and longevity [26,28,29].

Research on how society evaluates older doctors is lacking. The few studies that have been carried out examined patients’ preferences regarding the age of their physicians [30-33]. Although older physicians are commonly deemed more patient and reassuring [30,31], some believe they are more susceptible to dispensing a lower quality of care than their younger colleagues [32]. A recent study found that patients cared for by older practitioners had higher mortality rates than those treated by younger ones [34]. In the legal sphere, minimal attention has been devoted to examining the ways in which older lawyers are stereotyped. That said, prior scholarship has hinted at both the opportunities and challenges posed by older attorneys. On one hand, there has been discourse on the need to leverage the skills of older attorneys, be it by allowing them to register for emeritus pro bono status [35] or by offering them opportunities to train younger attorneys [36]. On the other hand, there have been fears that older attorneys’ ability to continue their practice may be hampered by cognitive changes [35].

We test 2 hypotheses. First, to determine how age affects portrayals of doctors and lawyers in the media, we compare the difference in sentiments toward older and younger doctors, as well as older and younger lawyers. We hypothesize that the media will place a premium on youth in the medical profession where physical dexterity—a trait typically associated with younger people [37]—is prized across a range of specialties [38,39]. Specifically, we hypothesize that sentiments toward younger doctors will be more positive than toward older doctors over 21 decades in the media (hypothesis 1). Second, we hypothesize that the media will place a premium on old age in the legal profession due to its inherent association with experience—a quality of paramount importance in the legal domain [35]. Specifically, we hypothesize that sentiments toward older lawyers will be more positive than toward younger lawyers over the same period (hypothesis 2).

## Methods

### Data Set

Following earlier work [10,40-42], we created the largest historical corpus—comprising 600 million words—of American media publications spanning 210 years (1810 to 2019) by merging the Corpus of Historical American English (COHA) from 1810 to 2009 with the Corpus of Contemporary American English (COCA) from 2010 to 2019 [43]. The combination of both corpora formed the largest structured historical English corpus with over 150,000 texts collected from newspapers, magazines, fiction, and nonfiction. Publications were extracted from major news outlets such as the *New York Times*, *Wall Street Journal*, and *USA Today*, as well as smaller ones like the *Atlantic Journal Constitution* and the *San Francisco Chronicle*. Material that was published at some point throughout the 210 years but that has ceased publication was included in the data set. The media represents a significant platform for examining age stereotypes as it possesses tremendous power to shape public opinion [4-6].

### Target Nouns to Measure Occupation and Age × Occupation Stereotypes

#### Occupation

The Harris Poll [44] listed doctors (physicians) and lawyers (attorneys) as some of the most respected professions. Prior studies have also found that doctors (physicians) and lawyers (attorneys) are seen as some of the most functionally significant occupations in society [45]. To ensure the relevance and applicability of our findings, the terms we selected for analysis are those frequently used in American media to describe professionals in the legal and medical fields. These terms are “doctor,” “physician,” “lawyer,” and “attorney.”

#### Old Age × Occupation

The descriptors or adjectives for the following terms were compiled: “old doctors,” “older doctors,” “old physicians,” “older physicians,” “old lawyers,” “older lawyers,” “old attorneys,” and “older attorneys.” We took into consideration the fact that older workers may be referred to by other adjectives such as “aging,” “elder,” “elderly,” and “aged.” However, we used “old” and “older” as they evidenced the highest prevalence in the data set.

#### Youth × Occupation

The descriptors or adjectives for the following terms were compiled: “young doctors,” “younger doctors,” “young physicians,” “younger physicians,” “young lawyers,” “younger lawyers,” “young attorneys,” and “younger attorneys.” Although younger workers may be referred to by other adjectives including “junior,” we used “young” and “younger” to exclude words describing workers of a lower status.

### Selection of Descriptors and Sentiment Scoring

The top descriptors that co-occurred most frequently—referred to as “collocates”—with each term were compiled per decade for 210 years based on the following inclusion criteria: (1) The collocate was present within 6 words before or after the target word (lexical proximity). Articles such as “the” and “a” were not included in the 6-word lexical span. If the target noun was the first word of a sentence, collocates from the preceding sentence were excluded. (2) The collocate referred to an older or younger person specifically (relevant context). (3) There was a mutual information score of 1.5 and above, which suggests semantic bonding, meaning that the collocate has a stronger association with the particular synonym than other words in the corpus [46]. We use the following formula to calculate the mutual information score:



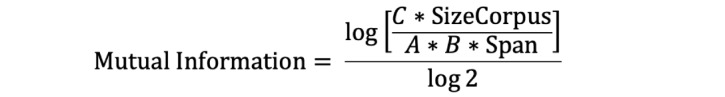



“A” indicates the possibility of the target word A appearing, which is calculated by the frequency of the target word. “B” indicates the possibility of the collocate B appearing, which is calculated by the frequency of word B. “C” indicates the possibility of “A” and “B” appearing together, which is calculated by the frequency of collocate B appearing near the target word A. “SizeCorpus” refers to the size of the corpus or the number of words. Span is the span of words, that is, if there are 6 words to the left and 6 words to the right of the target word, span=12; log (2)=0.30103. This is an application of concordance analysis called “psychomics”, which has been used in past literature to analyze societal stereotypes [10,47-50]. This rigorous process culminated in 478,452 collocates (descriptors or adjectives).

To test both hypotheses, each collocate was rated using a sentiment engine on a scale from 1 (very negative) to 5 (very positive). This has proven to be a valid and reliable method of measuring words associated with age stereotypes [51] and follows previous corpus-based analyses [52]. Very negative collocates were rated 1 (eg, “frail” and “burden”), neutral collocates were rated 3 (eg, “transport” and “society”), and very positive collocates were rated 5 (eg, “venerable” and “cheerful”). For every noun per decade, we tabulated a mean score that was then weighted (by the number of times the respective noun appeared in that decade) to determine the respective sentiment score.

### Analytic Strategy

Hypothesis 1 states that sentiments toward younger doctors in the media are more positive than toward older ones, while hypothesis 2 states that sentiments toward older lawyers in the media are more positive than toward younger lawyers. Both hypotheses were tested by analyzing the respective sentiment trends over 210 years (1810 to 2019) and thereafter determining whether the respective slopes were significantly different. Topic modeling was conducted on the descriptors of each occupation (eg, older doctor) in both the 1800s and 1900s using latent Dirichlet allocation (LDA) [53]. By probabilistically grouping words into topics, LDA identifies latent topics and clusters of words that co-occur frequently [53]. All data preprocessing, text analytics, and statistical analyses were done in Python 3.7 and OriginLab Corporation’s OriginPro 2019b (OriginLab Corporation).

### Ethical Considerations

No ethical approval was sought for this study due to the publicly available and nonidentifiable nature of the data. Moreover, this study was exempted from ethics review as it involved a secondary analysis of publicly available material.

## Results

### Youth Premium in Depictions of Doctors in the Media (Hypothesis 1)

**Figure 1:**
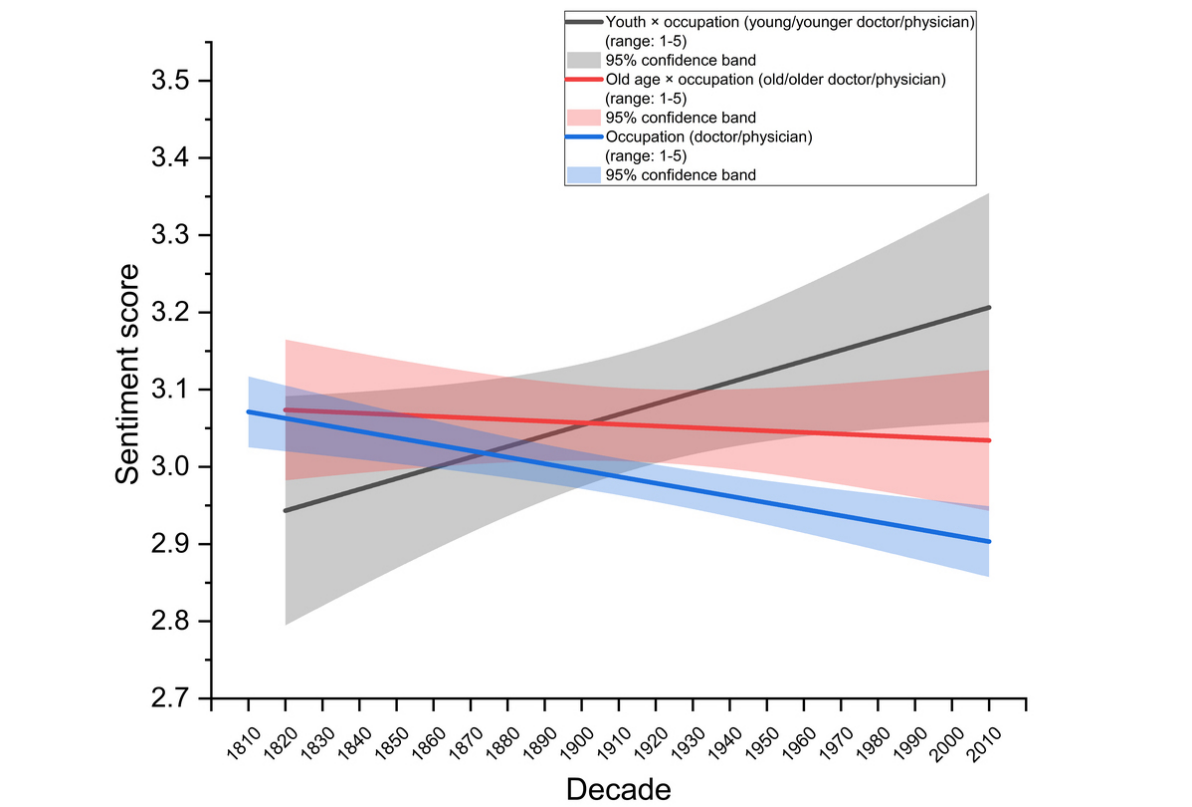
The youth premium in the medical profession over 2 centuries from 1810 to 2019. Portrayals of younger doctors or physicians in the media became 10% more positive across 21 decades, while those of older doctors became 1.4% more negative, though this latter trend was not statistically significant. The shaded regions refer to the 95% confidence band of the respective lines. The sentiment score (y-axis) ranges from 1 (most negative) to 5 (most positive).

We tested whether there was a youth premium in depictions of doctors in the media. As hypothesized, younger doctors or physicians evidenced more positive societal sentiments than older doctors or physicians over 21 decades from 1810 to 2019. Younger doctors enjoyed a 10% increase in positive portrayals over 210 years (β=0.01385, *P*=.04), while older doctors experienced a 1.4% decline in sentiments over the same period, though this trend was not statistically significant (Figure 1). The difference across both slopes achieved statistical significance, *F*_1,36_=4.5602, *P*=.04, providing support for hypothesis 1.

### Old Age Premium in Depictions of Lawyers in the Media (Hypothesis 2)

**Figure 2:**
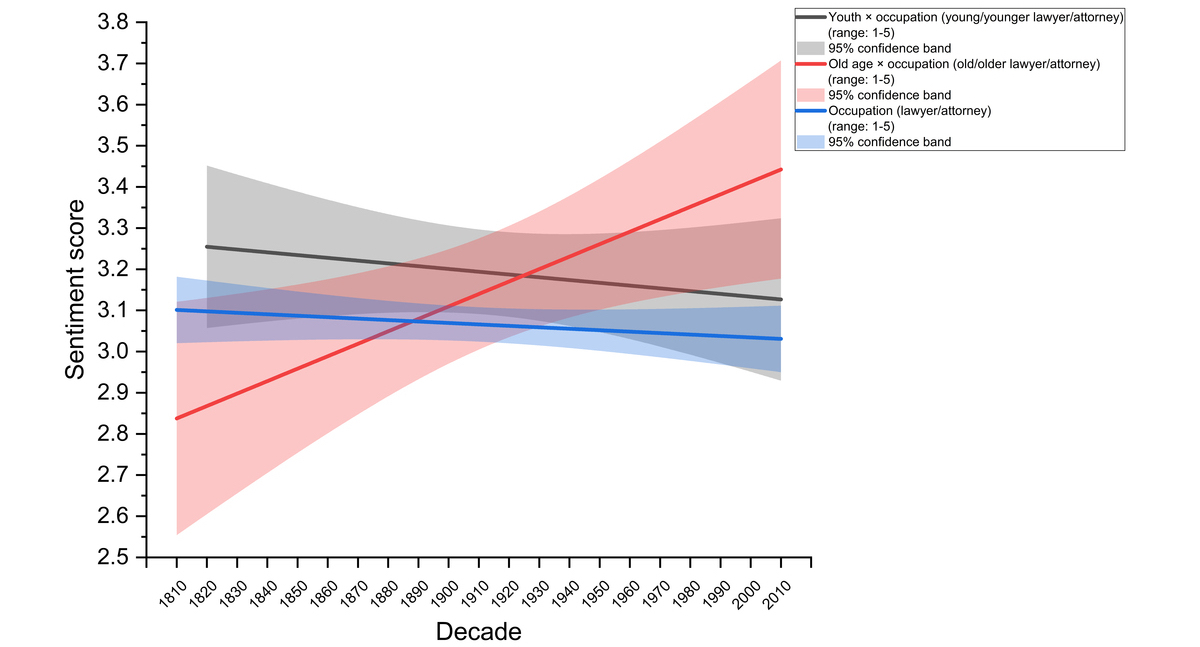
The old-age premium in the legal fraternity over 2 centuries from 1810 to 2019. From 1810 to 2019, portrayals of older lawyers in the media became 22.6% more positive, compared with 4.3% more negative for younger lawyers. The shaded regions refer to the 95% confidence band of the respective lines. The sentiment score (y-axis) ranges from 1 (most negative) to 5 (most positive).

We tested whether there was a premium of old age in depictions of lawyers in the media. As hypothesized, older lawyers or attorneys evidenced more positive sentiments compared with younger lawyers or attorneys across 21 decades from 1810 to 2019. Older lawyers experienced a 22.6% increase in positive portrayals over 21 decades (β=0.0302, *P*=.02). Conversely, younger lawyers experienced a 4.3% decline in sentiments over the same period (β=–0.00673, *P*≤.001). These results reflect an age premium for lawyers where older lawyers are portrayed more positively relative to younger ones (Figure 2). The difference across both slopes reached statistical significance, *F*_1,34_=7.085, *P*=.01, supporting hypothesis 2.

### Summary of Insights from Topic Modeling

Topics were generated for old age × occupation and youth × occupation framing in the 1800s and 1900s. In the 1800s, narratives on younger doctors revolved around their participation in rural health care. In the 1900s, the focus shifted to their mastery of new medical technologies. Unlike younger doctors, there was no marked change in narratives surrounding older doctors from the 1800s to 1900s, though less attention was paid to their skills in the 1900s. Narratives on younger lawyers in the 1800s described them as having limited experience. In the 1900s, there was more focus on courtroom affairs. In both the 1800s and 1900s, narratives on older lawyers emphasized their prestige. This emphasis was more apparent in the 1900s as their seniority and experience became increasingly valuable in navigating the growing complexity of court cases. These results are summarized in Table 1.

**Table 1:** Summary of narratives in the media for old age × occupation (doctors or physicians and lawyers or attorneys) and youth × occupation (doctors or physicians and lawyers or attorneys) in the 1800s and 1900s.

Framing	1800s	1900s
Youth × occupation framing (doctors or physicians)	• Medical degree • Interactions with patients • Religion and medicine • Sickness and death in the hospital • Diagnosis and prescription • Rural health care	• Medical knowledge • High-quality care • Sickness and death in the hospital • Treatment and recovery • Financial woes • Consultations and medical appointments
Old age × occupation framing (doctors or physicians)	• Skill and expertise • Visits to the hospital • Religion and medicine • Sickness and death in the hospital	• Experience • Other occupations • Diagnosis and prescription • Sickness and death in the hospital • Surgery • Bedside manner • Royal visits
Youth × occupation framing (lawyers or attorneys)	• Prestige • Law and religion • Judicial district • Prosecution • Limited experience	• Courtroom affairs • Trial and prosecution • Politics • Lawsuits against politicians • Legal fees • Other occupations
Old age × occupation framing (lawyers or attorneys)	• Prestige • Legal guidance • Prosecution • Legal fees	• Seniority and experience • Courtroom proceedings • Legal guidance • Bar • Lawsuits against politicians • Family law

### Youth × Occupation Framing (Doctors or Physicians) in the 1800s

Topic 1 consists of terms pertaining to obtaining a “medical degree” (“college,” “degree,” and “professor”). Topic 2 touches on “interactions with patients” (“laugh,” “smile,” and “boy”). Collocates alluding to “religion and medicine” are in topic 3 (“reverend,” “pray,” and “advise”). Topic 4 is about “sickness and death in the hospital” (“sick,” “dead,” and “fever”) while topic 5 is about “diagnosis and prescription” (“prescribe,” “medicine,” and “care”). Topic 6 focuses on “rural healthcare” (“village,” “horse,” “ride,” and “inquire”).

### Youth × Occupation Framing (Doctors or Physicians) in the 1900s

Collocates in topic 1 paint younger physicians as having up-to-date “medical knowledge” and being proficient in the use of newer medical technologies (“treatment,” “medical,” “informatics,” and “technology”). Those in topic 2 hint at how younger doctors dispense “high-quality care” (“better” and “care”). Topic 3 is about “sickness and death in the hospital” (“sick,” “disease,” “and “die”). Topic 4 dwells on “treatment and recovery” (“treatment,” “cure,” and “best”). Topic 5 looks at the “financial woes” confronted by some patients (“shoulder,” “worry,” “bill,” and “enough”) and topic 6 at “consultations and medical appointments” (“consult,” “advise,” “and “visit”).

### Old Age × Occupation Framing (Doctors or Physicians) in the 1800s

Topic 1 describes the “skill and expertise” of older doctors (“skill” and “experience”). Topic 2 covers “visits to the hospital” (“visit,” “consult,” and “opinion”). Topic 3 makes reference to “religion and medicine” (“divinity,” “village,” and “medicine”) and topic 4 to “sickness and death in the hospital” (“die” and “trouble”).

### Old Age × Occupation Framing (Doctors or Physicians) in the 1900s

Topic 1 deals with the wealth of “experience” of older doctors (“experience” and “practitioner”). Comparisons of doctors with “other occupations” (“lawyer,” “surgeon,” and “dentist”) are in topic 2. Topic 3 is about “diagnosis and prescription” (“prescription,” “drug,” and “diagnosis”) and topic 4 is about “sickness and death in the hospital” (“disease” and “dead”). Topic 5 pertains to “surgery” (“operation,” “perform,” and “abortion”). Topic 6 looks at “bedside manner” (“laugh” and “smile”) and topic 7 involves “royal visits” (“royal,” “visit,” and “news”).

### Youth × Occupation Framing (Lawyers or Attorneys) in the 1800s

Words in topic 1 portray the legal profession as one of “prestige” (“eminent,” “distinguished,” and “prominent”). Those in topic 2 concern “law and religion” (“clergyman,” “judge,” and “divine”). Topic 3 features terms related to the “judicial district” (“judicial,” “district,” “jury,” and “statesman”) and topic 4 features terms related to “prosecution” (“prosecute” and “defend”). Collocates in topic 5 imply that younger attorneys have “limited experience” (“less,” “experience,” and “knowledge”).

### Youth × Occupation Framing (Lawyers or Attorneys) in the 1900s

Topic 1 comprises terms related to “courtroom affairs” (“affairs,” “drama,” “judge,” and “court”). Topic 2 circles around issues regarding “trial and prosecution” (“plead,” “charge,” and “criminal”). Matters regarding “politics” dominate topic 3 (“republican,” “democratic,” and “senate”). Specific “lawsuits against politicians” are in topic 4 (“settlement,” “lawsuit, “allegation,” and “complaint”). Topic 5 focuses on “legal fees” (“legal,” “insurance,” “interest,” and “cost”) and topic 6 compares the legal profession to “other occupations” (“accountant,” “banker,” “doctor,” and “teacher”).

### Old Age × Occupation Framing (Lawyers or Attorneys) in the 1800s

Topic 1 depicts older lawyers as having a certain level of “prestige” (“eminent” and “respectable”). Topic 2 revolves around “legal guidance” (“opinion” and “advice”). Topic 3 covers “prosecution” (“prosecute,” “evidence,” and “judge”) and topic 4 contains terms related to “legal fees” (“interest,” “dollar,” and “fee”).

### Old Age × Occupation Framing (Lawyers or Attorneys) in the 1900s

The idea that older attorneys have more “seniority and experience” is in topic 1 (“senior,” “experience,” “successful,” and “complex”). Topic 2 discusses matters regarding “courtroom proceedings” (“judge,” “defendant,” and “trial”). Topic 3 is related to “legal guidance” (“hire” and “represent”). Topic 4 is about the “bar” (“association” and “conference”) and topic 5 is about “lawsuits against politicians” (“file,” “governor,” and “republican”). Topic 6 involves “family law” (“divorce,” “witness,” and “son”).

## Discussion

In this study, we compared how sentiments toward older and younger doctors and lawyers have changed in the media over the last 2 centuries. Findings reveal that media outlets have placed a premium on youth in the medical enterprise but on old age in the legal fraternity.

Narratives on younger doctors in the media are more positive than on older doctors. Additionally, while narratives on older doctors have become more negative, narratives on younger doctors are experiencing the reverse trend. One possible reason for these trends is the rise of new medical technologies and techniques that younger doctors are usually assumed to be more conversant with. Moreover, medical practitioners are said to be the most proficient in the years immediately after completing residency training. There is a common belief that doctors further from training may rely on out-of-date clinical evidence and not adhere as rigidly to evidence-based guidelines as younger doctors [33]. As seen in our LDA results, from the 1900s, the media began emphasizing the merits of younger physicians by highlighting their up-to-date medical knowledge. Although there were portrayals of older doctors as being skilled in the 1800s, this changed in the 1900s, during which more attention was dedicated to their experience and bedside manner. Thus, there may have been a subconscious association of youth with medical competence in the media.

Narratives on older lawyers in the media are more positive than those on younger lawyers. While narratives on older lawyers have become more positive, the trend is the opposite for younger lawyers. Younger attorneys may be seen as more enthusiastic and willing to learn, but they may also be viewed as lacking the expertise of their older and more seasoned counterparts. The increasing specialization of the legal profession over time [54] may have resulted in age becoming more highly valued in the field. Older attorneys may be seen as possessing a deeper understanding of legal precedents and a greater ability to navigate the complexities and intricacies of legal matters [35]. Our LDA results uncovered that since the 1900s, older lawyers have been depicted in the media as having a wealth of expertise, which may render them better able to provide valuable legal guidance to clients and colleagues alike.

The issue of youth-directed ageism is beyond the ambit of this study. Nevertheless, it cannot go unacknowledged that narratives on younger lawyers have become increasingly negative. Over the years, clarion calls have been sounded for bullying in the legal profession to end. In 2018, the International Bar Association surveyed 7000 lawyers from 135 countries on the topic of bullying and sexual harassment in the profession [55]. Results from the survey lent empirical support to claims about the rampancy of bullying and sexual harassment in the legal enterprise, which may not be particularly shocking in view of the adversarial, hierarchical, and hypercompetitive nature of the job [55]. Younger legal professionals were found to be disproportionately affected by bullying, and respondents from the United States reported higher rates of both bullying as well as sexual harassment than the global average [55]. This may account for the increase in negativity associated with narratives on younger attorneys.

In line with social constructionism [4], agenda-setting [5,6], and cultivation theories [7], the media is instrumental in shaping public perceptions of older adults. Depictions of younger physicians as being well acquainted with the newest medical technologies, though particularly important as they begin their medical careers, may come at the expense of older doctors. The public may be conditioned to view younger doctors as superior to older ones, which could consequently foment ageist stereotypes. The fact that depictions of older lawyers have grown more positive over time is heartening as it shows that older lawyers are being celebrated for their seniority and expertise.

The finding that an age premium exists in depictions of lawyers but not in depictions of doctors is interesting. It may be that the age of a physician is perceived as more important as work in the medical setting may have life-threatening consequences. Indeed, scholars have contended that older physicians’ waning physical skills may pose major surgical risks [38,39]. Such risks may be thought to outweigh the potential lessons to be imparted by older doctors. Physical dexterity has comparatively little—if any—bearing on whether one is able to perform one’s legal duties effectively, which may explain the differing results between portrayals of older doctors and lawyers.

This study yields some important insights. First, it is important that narratives on older adults in the media accurately mirror the contributions of older adults to society. More attention should be dedicated to the immense value they add to the workplace and economy in order that negative stereotypes are progressively removed from one’s cognitive repertoire. Just as the media has highlighted the strengths of older lawyers, it may be worthwhile to increase media coverage of the unique strengths of older doctors and what they can bring to patient care. Media campaigns could be held to promote positive images of older doctors. Such campaigns could feature interviews with these doctors, details of their achievements, and testimonials from patients who have received exceptional care from them. Additionally, media outlets could offer training programs for journalists to sensitize them to the issue of ageism and to equip them with guidelines on writing about older doctors tactfully. To avoid perpetuating ageist stereotypes and to promote better psychological well-being among older persons [26], it is crucial to ensure accurate depictions of older professionals in the media. As only a small fraction of older professionals are covered in the media, the few depictions that do exist hold significant influence in shaping public attitudes toward aging.

Second, our results indicate that depending on the occupation, old age may be deemed more valuable than youth. There is therefore a need to explore how the merits of old age—skill, knowledge, and experience—in these occupational contexts could be extended to the way the older population is viewed as a whole. To this end, it may be useful to encourage more interaction between older and younger people in the workplace. Age diversity may translate into advantages in both human and social capital [56] as knowledge is passed from young to old and old to young [57]. Relatedly, it is imperative that older persons are eased into the retirement phase and not tossed into it unceremoniously. Opportunities to continue contributing to the workplace—such as through mentorship programs—should be provided so as to harness the treasury of skills and knowledge of this cohort. This will also allow older persons to transition more seamlessly into the next phase of life.

This study has various limitations. First, we acknowledge that an emphasis on occupational roles may wind up perpetuating the notion that older people are to be valued only if they remain economically productive [58]. However, our findings reveal that old age may evoke positive stereotypes in certain occupational contexts, which has implications on how negative stereotypes of older adults can be eliminated. Second, as only 2 occupational roles were used for analysis, the differences in portrayals of older and younger workers in other occupational settings remain an uncharted area of research. Furthermore, the occupations considered in this study are generally considered to be prestigious. Future scholarship could examine whether old age is valued in the context of blue-collar occupations. Third, our search only included the 2 professions as umbrella terms without specifying the various specialties within each profession, such as cardiologists, ophthalmologists, criminal defense prosecutors, and tax attorneys. Thus, our study only lays out in broad strokes how older lawyers and doctors are depicted in the media. Future studies could address this limitation by delving into the various subcategories.

Fourth, the data set used in this study only comprises American sources. The meanings ascribed to age and to occupations are likely to differ across cultural contexts. For instance, the idea of the American dream may have had an impact on the portrayal of the legal profession since it could be tied closely to the notion of wealth and success. A study that investigates how portrayals of older doctors and lawyers vary across cultures may hence be worth pursuing. Finally, it is important to acknowledge that LDA may not account for the context in which the words are used, making it difficult to discern whether ageist portrayals are propagated by journalists or other sources. In addition, LDA may not detect nuances in language such as tone, sarcasm, and irony. Further research is therefore required to determine the generalizability of our findings. Other directions for future study include an analysis of how narratives pertaining to select occupations have unfolded since the 2000s. It is likely that topics related to artificial intelligence and automation will emerge in these narratives. Surveys [59], interviews [60], and big data analytics [61,62] could also be used to explore the types of stereotypes linked to professionals of different age groups.

### Conclusions

In seeking to eradicate ageism, perhaps a pressing question is not so much whether this phenomenon exists, but rather in what situations it manifests itself and to what degree. This study has demonstrated that depending on the occupation, one’s age may either be seen as an asset or a liability. Moving forward, effort must be expended to ensure that older professionals are recognized for their wealth of knowledge and skills. Failing to capitalize on the merits of an aging workforce could ultimately be a grave disservice not only to older adults but to society in general.

## Abbreviations

COCA: Corpus of Contemporary American English
COHA: Corpus of Historical American English
LDA: latent Dirichlet allocation

## Data Availability

Data are publicly available at English-Corpora.org [63].
